# T25. ALTERATIONS OF PLASMA PHOSPHOLIPID FATTY ACID PROFILES ARE ASSOCIATED WITH LOCAL BRAIN STRUCTURAL ABNORMALITIES IN NEUROLEPTIC NAïVE FIRST-EPISODE PSYCHOSIS PATIENTS

**DOI:** 10.1093/schbul/sby016.301

**Published:** 2018-04-01

**Authors:** Kerstin Langbein, Christian Fleischer, Katrin Kuhnt, Christian Gaser, Stefan Smesny

**Affiliations:** 1 Jena University Hospital; 2 Friedrich-Schiller-University Jena

## Abstract

**Background:**

Alterations in brain structure are among the most robust biological findings in schizophrenia. Also changes in fatty acid profiles including reduced levels of polyunsaturated fatty acids (PUFA) are well replicated in schizophrenia. Being essential for neurodevelopmental processes and structural plasticity and remodeling, alterations of PUFA metabolism might be also associated with the occurrence of brain structural abnormalities. To investigate this assumption in vivo we examined the interrelation of PUFA profiles and brain structure in neuroleptic naïve first-episode psychosis (FEP) patients and healthy controls (HC) matched for age and gender.

**Methods:**

High-resolution T1-weighted 3T MRI were acquired from 29 FEP patients (age 26.4 ± 5.3y; 13 females/16 males) and 31 HC (age 25.1 ± 4.7y; 14 f/17 m). Fatty acid profiles were analyzed using gas chromatography in the plasma phospholipid (PL) fraction that is rather independent of recent fat consumption, and that potentially indicates PUFA availability in phospholipid structural components essential in the brain. To investigate brain structural abnormalities in FEP and effects of illness on interactions between fatty acid profiles and local grey (GM) or white (WM) matter density, voxel-based morphometry (VBM) with the computational anatomy toolbox (CAT12) was used.

**Results:**

VBM analyses revealed a reduction of GM in FEP in left frontal operculum, left middle frontal gyrus, left superior frontal gyrus and bilateral temporal gyrus (TFCE, FWE<0.05). The group comparisons of fatty acid profiles in the PL fraction showed reduced omega-6 PUFA and MCFA (C10-C14) levels in FEP patients. Interaction analyses revealed an influence of illness on the association between omega-6 PUFA and GM density at the left supramarginal gyrus/left postcentral gyrus and left superior temporal gyrus (TFCE, FWE<0.05) with a regression slope FEP patients > HC. For MCFA interaction analyses revealed effects of illness on associations with GM density in the bilateral superior medial frontal gyrus and left anterior cingulate gyrus with a regression slope HC > FEP patients.

**Discussion:**

Our results support the notion that the availability of PUFA and MCFA potentially affects brain structural development and remodeling. They also support the notion of PUFA and phospholipids metabolism as a biochemical basis to create early prevention and intervention strategies, e.g. by PUFA supplementation.

